# Supplementary material for: Multiple omics analyses and experiments validation identify PRDX3 as a biomarker of prognosis and antioncogene in kidney clear cell carcinoma
Source: PLoS One. 2026 Mar 16;21(3):e0345095. doi: 10.1371/journal.pone.0345095 (PMC12991247; doi:10.1371/journal.pone.0345095)
Supplement: S1 Table — (DOCX) [file pone.0345095.s001.docx]

**S1 Table** Lists of abbreviations

| **Abbreviation** | **Full name** |
| --- | --- |
| ACC | Adrenocortical carcinoma |
| BLCA | Bladder Urothelial Carcinoma |
| BRCA | Breast invasive carcinoma |
| CESC | Cervical squamous cell carcinoma and endocervical adenocarcinoma |
| CHOL | Cholangiocarcinoma |
| COADREAD | Colon adenocarcinoma/Rectum adenocarcinoma Esophageal carcinoma |
| DLBC | Lymphoid Neoplasm Diffuse Large B-cell Lymphoma |
| ESCA | Esophageal carcinoma |
| GBM | Glioblastoma multiforme |
| HNSC | Head and Neck squamous cell carcinoma |
| KICH | Kidney Chromophobe |
| KIRC | Kidney renal clear cell carcinoma |
| KIRP | Kidney renal papillary cell carcinoma |
| LAML | Acute Myeloid Leukemia |
| LGG | Brain Lower Grade Glioma |
| LIHC | Liver hepatocellular carcinoma |
| LUAD | Lung adenocarcinoma |
| LUSC | Lung squamous cell carcinoma |
| MESO | Mesothelioma |
| OV | Ovarian serous cystadenocarcinoma |
| PAAD | Pancreatic adenocarcinoma |
| PCPG | Pheochromocytoma and Paraganglioma |
| PRAD | Prostate adenocarcinoma |
| READ | Rectum adenocarcinoma |
| SARC | Sarcoma |
| SKCM | Skin Cutaneous Melanoma |
| STAD | Stomach adenocarcinoma |
| STES | Stomach and Esophageal carcinoma |
| TGCT | Testicular Germ Cell Tumors |
| THCA | Thyroid carcinoma |
| THYM | Thymoma |
| UCEC | Uterine Corpus Endometrial Carcinoma |
| UCS | Uterine Carcinosarcoma |
| UVM | Uveal Melanoma |
| PRDXs | Peroxiredoxins |
| TCGA | the Cancer Genome Atlas program |
| OS | Overall Survival |
| PFI | Progression-free interval |
| DSS | disease specific survival |
| DFI | disease-free interval |
| TMB | Tumor mutation burden |
| MSI | Microstatellite instability |
| LRRK2 | Leucine Rich Repeat Kinase 2 |
| HSPD1 | Heat Shock Protein Family D (Hsp60) Member 1 |
| OPA1 | OPA1 Mitochondrial Dynamin Like GTPase |
| PARK7 | Parkinsonism Associated Deglycase |
| DLD | Dihydrolipoamide Dehydrogenase |
| SNCA | Synuclein Alpha |
| DHRS4 | Dehydrogenase/Reductase 4 |
| RPS6KC1 | Ribosomal Protein S6 Kinase C1 |
| UCHL5 | Ubiquitin C-Terminal Hydrolase L5 |
| KRAS | KRAS Proto-Oncogene, GTPase |
| IL6 | Interleukin 6 |
| JAK | Janus Kinase 2 |
| STAT3 | Signal Transducer And Activator Of Transcription 3 |
| IL2 | Interleukin 2 |
| STAT5 | Signal Transducer And Activator Of Transcription 5 |
| TNFRSF | TNF Receptor Superfamily Member |
| NT5E | 5'-Nucleotidase Ecto |
| RAET1E | Retinoic Acid Early Transcript 1E |
| CD274 | Programmed Cell Death 1 |
| CCND | Cyclin D2 |
| MMP9 | Matrix Metallopeptidase 9 |
| CCL20 | C-C Motif Chemokine Ligand 20 |
