# Supplementary material for: Multiple omics analyses and experiments validation identify PRDX3 as a biomarker of prognosis and antioncogene in kidney clear cell carcinoma
Source: PLoS One. 2026 Mar 16;21(3):e0345095. doi: 10.1371/journal.pone.0345095 (PMC12991247; doi:10.1371/journal.pone.0345095)
Supplement: S2 Table — (DOCX) [file pone.0345095.s002.docx]

**S2 Table** The list of primers used in the reactions for reverse transcription qPCR

| Gene |  | Sequence |
| --- | --- | --- |
| PRDX3 | Forward | 5’-CCAACTTTGTGCCAACCGGTCGCCACCATGGCGGCAGCTGCGGGAAG-3‘ |
|  | Reverse | 5‘-AATGCCAACTCTGAGCTTCTGATGGACCTTCTCAAAGTACTC-3‘ |
| PPARA | Forward | 5‘-CTATAATTTGCTGTGGAGATCGGC-3’ |
|  | Reverse | 5‘-GGATGGTTGCTCTGCAGGT3’ |
| CCND | Forward | 5’CGTGGCCTCTAAGATGAAGG-3’ |
|  | Reverse | 5’-CCACTTGAGCTTGTTCACCA-3’ |
| CCL20 | Forward | 5’-GCTCCTGGCTGCTTTGATGT-3’ |
|  | Reverse | 5’-TTGATGTCACAGCCTTCATTGG-3’ |
| MMP9 | Forward | 5’-CAGTCCACCCTTGTGCTCTT-3’ |
|  | Reverse | 5’-ATTTCGACTCTCCACGCATC-3’ |
| Bax | Forward | 5’-CCCGAGAGGTCTTTTTCCGAG-3’ |
|  | Reverse | 5’-CCAGCCCATGATGGTTCTGAT-3’ |
| BMF | Forward | 5’-TCTCTGCTGACCTGTTTGCC-3’ |
|  | Reverse | 5’-TCTGGGTAGCTTTGTCTTCCT-3’ |
| Bim | Forward | 5’-CATCATCGCGGTATTCGGTTC-3’ |
|  | Reverse | 5’-AAGGTTGCTTTGCCATTTGGT-3’ |
