# Supplementary figures and images for: Multiple omics analyses and experiments validation identify PRDX3 as a biomarker of prognosis and antioncogene in kidney clear cell carcinoma
Source: PLoS One. 2026 Mar 16;21(3):e0345095. doi: 10.1371/journal.pone.0345095 (PMC12991247; doi:10.1371/journal.pone.0345095)

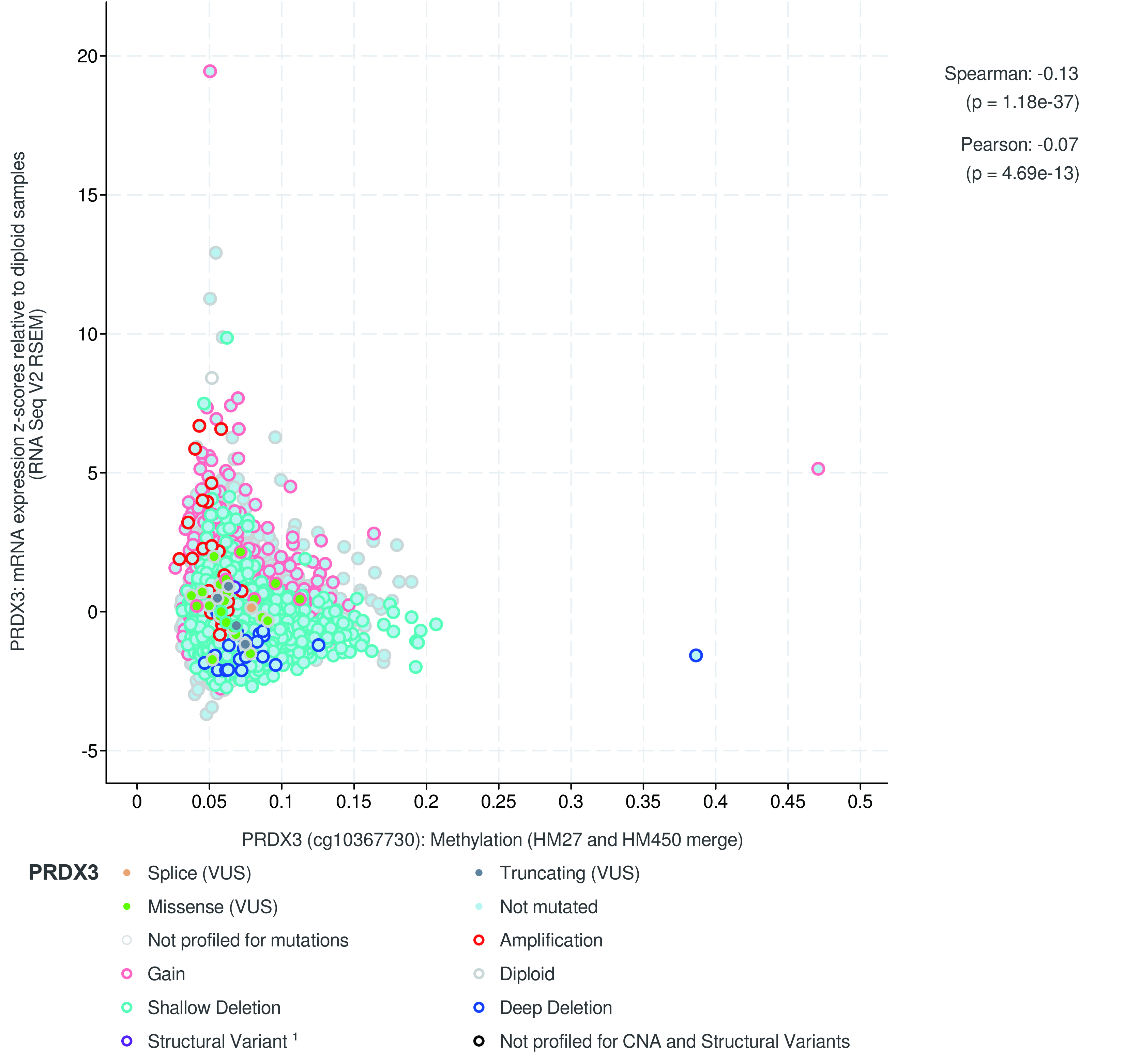

Supplement: S1 Fig — (TIF) [file pone.0345095.s003.tif]

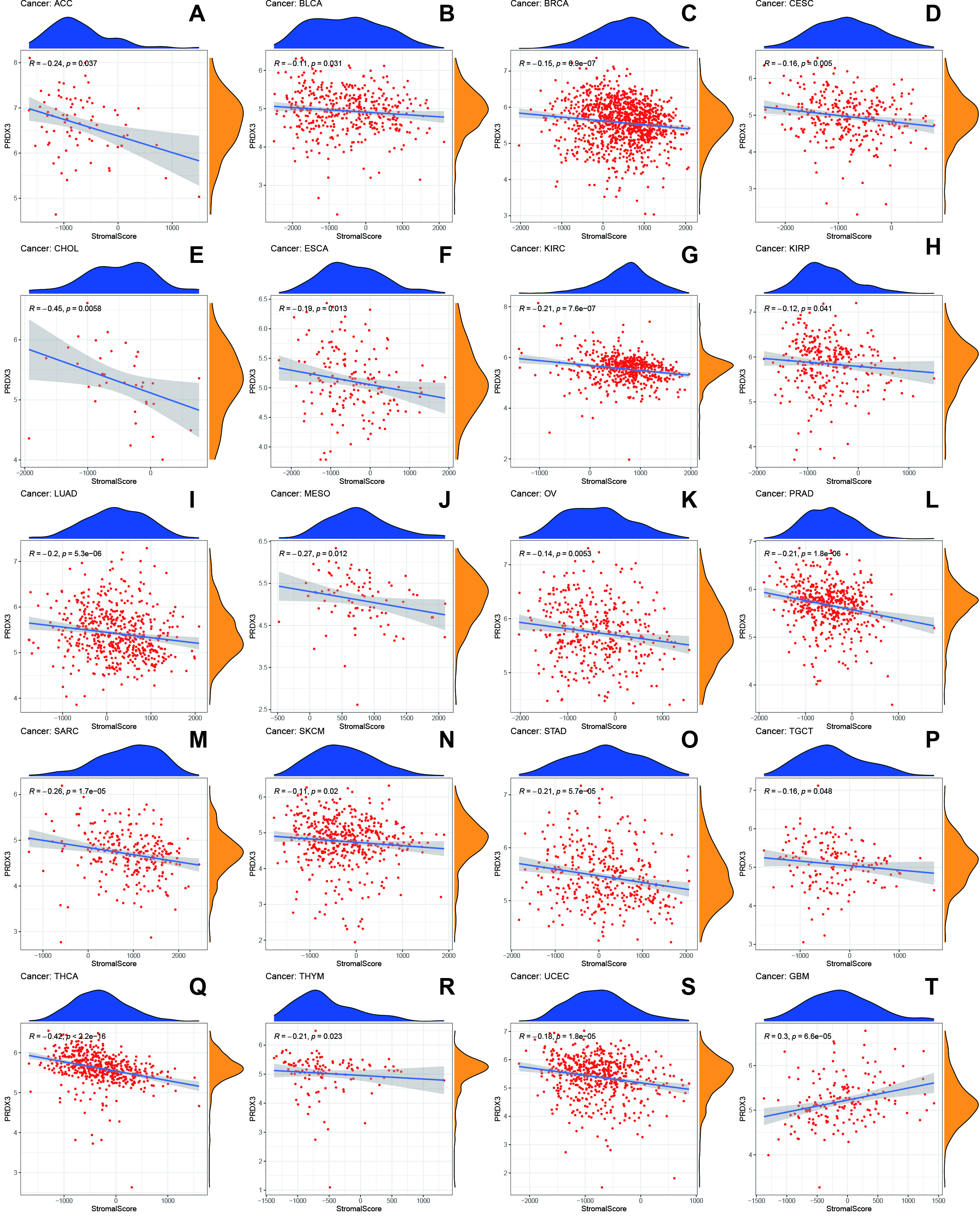

Supplement: S2 Fig — (TIF) [file pone.0345095.s004.tif]

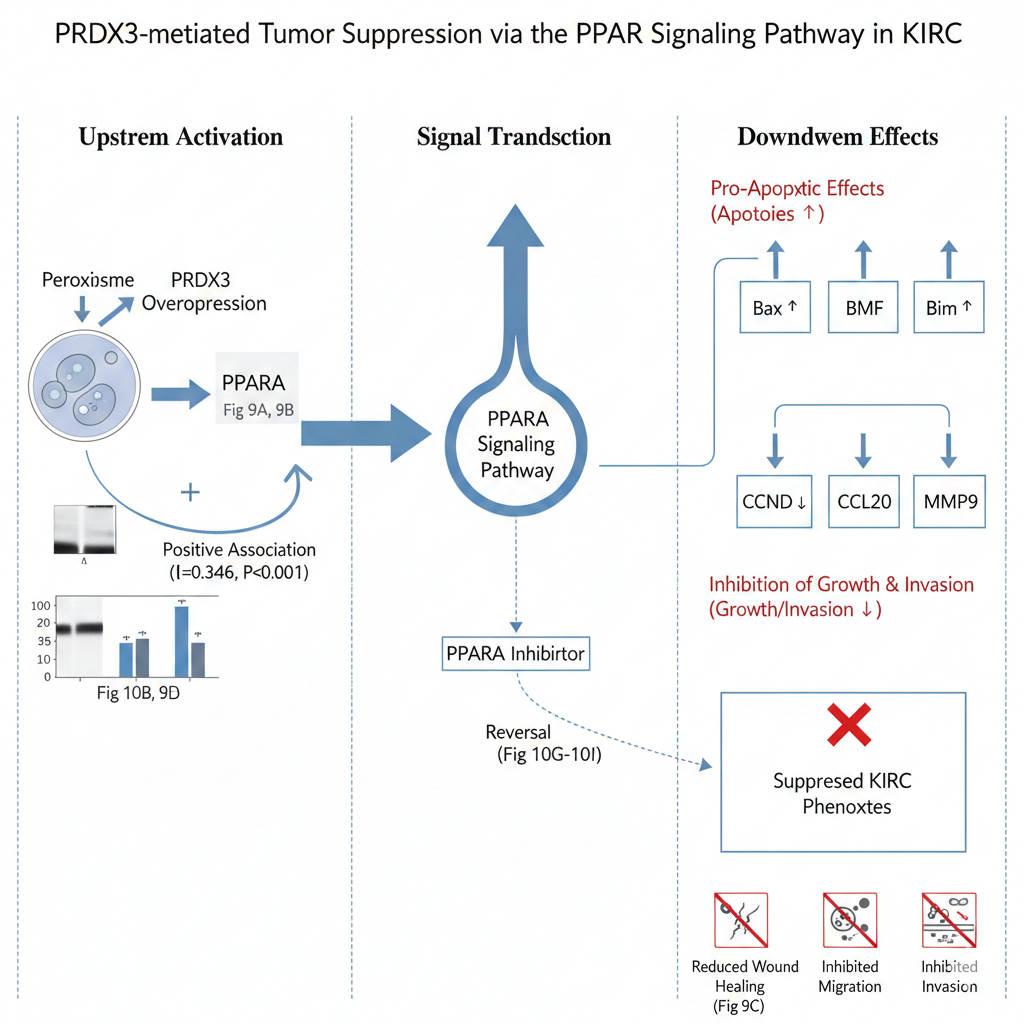

Supplement: S3 Fig — (TIF) [file pone.0345095.s005.tif]

**Figure 9A**

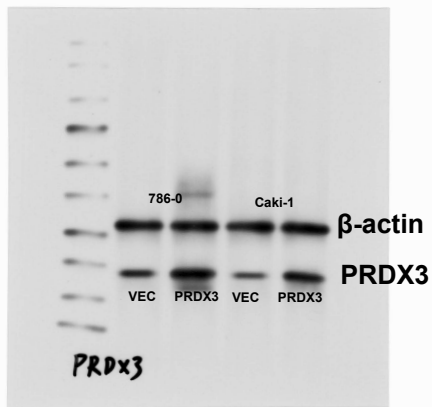

Figure 10C

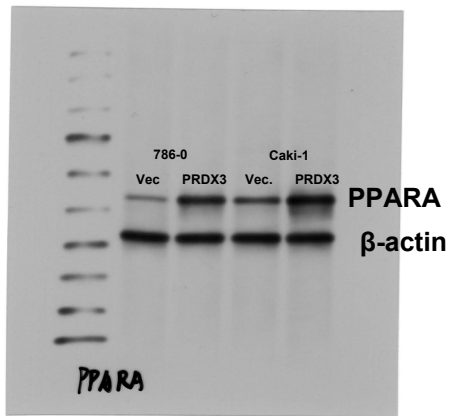

Supplement: S1 File — (PDF) [file pone.0345095.s006.pdf]
