# Supplementary material for: Multiple omics analyses and experiments validation identify PRDX3 as a biomarker of prognosis and antioncogene in kidney clear cell carcinoma
Source: PLoS One. 2026 Mar 16;21(3):e0345095. doi: 10.1371/journal.pone.0345095 (PMC12991247; doi:10.1371/journal.pone.0345095)
Supplement: Attachment — Submitted filename: Response to Reviewers.docx [file pone.0345095.s008.docx]

**Response to Editor and Reviewers**

**Response to Editorial comments**

Journal Requirements: When submitting your revision, we need you to address these additional requirements.

**Comment 1:** Please ensure that your manuscript meets PLOS ONE's style requirements, including those for file naming. The PLOS ONE style templates can be found at https://journals.plos.org/plosone/s/file?id=wjVg/PLOSOne_formatting_sample_main_body.pdf and https://journals.plos.org/plosone/s/file?id=ba62/PLOSOne_formatting_sample_title_authors_affiliations.pdf

**Response 1:** Yes, we have revised our manuscript according to these two samples, which meets PLOS ONE's style requirements.

**Comment 2:** PLOS requires an ORCID iD for the corresponding author in Editorial Manager on papers submitted after December 6th, 2016. Please ensure that you have an ORCID iD and that it is validated in Editorial Manager. To do this, go to ‘Update my Information’ (in the upper left-hand corner of the main menu), and click on the Fetch/Validate link next to the ORCID field. This will take you to the ORCID site and allow you to create a new iD or authenticate a pre-existing iD in Editorial Manager.

**Comment 2:** Yes, we have provided the ORCID iD for the corresponding author.

**Comment 3:** We note that Figure 1 in your submission contains copyrighted images. All PLOS content is published under the Creative Commons Attribution License (CC BY 4.0), which means that the manuscript, images, and Supporting Information files will be freely available online, and any third party is permitted to access, download, copy, distribute, and use these materials in any way, even commercially, with proper attribution. For more information, see our copyright guidelines: <http://journals.plos.org/plosone/s/licenses-and-copyright>. We require you to either (1) present written permission from the copyright holder to publish these figures specifically under the CC BY 4.0 license, or (2) remove the figures from your submission:1. You may seek permission from the original copyright holder of Figure 1 to publish the content specifically under the CC BY 4.0 license. We recommend that you contact the original copyright holder with the Content Permission Form (http://journals.plos.org/plosone/s/file?id=7c09/content-permission-form.pdf) and the following text: “I request permission for the open-access journal PLOS ONE to publish XXX under the Creative Commons Attribution License (CCAL) CC BY 4.0 (http://creativecommons.org/licenses/by/4.0/). Please be aware that this license allows unrestricted use and distribution, even commercially, by third parties. Please reply and provide explicit written permission to publish XXX under a CC BY license and complete the attached form.” Please upload the completed Content Permission Form or other proof of granted permissions as an "Other" file with your submission. In the figure caption of the copyrighted figure, please include the following text: “Reprinted from [ref] under a CC BY license, with permission from [name of publisher], original copyright [original copyright year].” 2. If you are unable to obtain permission from the original copyright holder to publish these figures under the CC BY 4.0 license or if the copyright holder’s requirements are incompatible with the CC BY 4.0 license, please either i) remove the figure or ii) supply a replacement figure that complies with the CC BY 4.0 license. Please check copyright information on all replacement figures and update the figure caption with source information. If applicable, please specify in the figure caption text when a figure is similar but not identical to the original image and is therefore for illustrative purposes only.

**Response 3:** Thank you. We have removed the copyrighted images and used our own figures in Figure 1.

**Comment 4:** PLOS ONE now requires that authors provide the original uncropped and unadjusted images underlying all blot or gel results reported in a submission’s figures or Supporting Information files. This policy and the journal’s other requirements for blot/gel reporting and figure preparation are described in detail at https://journals.plos.org/plosone/s/figures#loc-blot-and-gel-reporting-requirements and https://journals.plos.org/plosone/s/figures#loc-preparing-figures-from-image-files. When you submit your revised manuscript, please ensure that your figures adhere fully to these guidelines and provide the original underlying images for all blot or gel data reported in your submission. See the following link for instructions on providing the original image data: https://journals.plos.org/plosone/s/figures#loc-original-images-for-blots-and-gels. In your cover letter, please note whether your blot/gel image data are in Supporting Information or posted at a public data repository, provide the repository URL if relevant, and provide specific details as to which raw blot/gel images, if any, are not available. Email us at plosone@plos.org if you have any questions.

**Response 4:** Yes, we have provided original uncropped and unadjusted images underlying all blot or gel results reported in a submission’s figures and uploaded these images in the Supporting Information files (S1_File.pdf).

**Comment 5:** If the reviewer’s comments include a recommendation to cite specific previously published works, please review and evaluate these publications to determine whether they are relevant and should be cited. There is no requirement to cite these works unless the editor has indicated otherwise.

**Response 5:** Yes, we have review and evaluate these publications very carefully and make a cautious citation.

**Response to Reviewers**

**Response to Reviewer #1:**

This is an interesting pan-cancer plus mechanism paper with real experimental effort, but it still feel overextended and under-contextualized in places, so a major revision make sense to tighten the logic and strengthen the translational angle.
**Comment 1:** In the introduction, the background read solid but also a bit generic, and it would really benefit from stepping back and framing PRDX3 within the broader cancer biomarker landscape instead of jumping so fast into oxidative stress biology. Biomarker discovery is not just about association but about eventual utility, and this is where a short conceptual bridge help a lot. I strongly suggest anchoring the rationale by briefly discussing how discovery-stage biomarkers move toward prognosis, stratification, and eventually intervention, for example by citing “Bridging Discovery and Treatment: Cancer Biomarker,” which fit naturally here and give the reader a clearer sense of why PRDX3 matter beyond being another differentially expressed gene. That kind of framing would also make the pan-cancer scope feel more intentional rather than simply exhaustive.
**Response 1:** Thank you for your advice. We added the following descriptions in the introduction as follows: Therefore, clarifying tumor etiology and identifying clinically useful biomarkers and therapeutic targets remain critical for earlier detection, improved risk stratification, and better long-term outcomes. Importantly, biomarker research is not only about statistical association but also about eventual clinical utility. A biomarker is broadly defined as a measurable characteristic that reflects normal or pathogenic processes or responses to an intervention, and cancer biomarkers may serve diagnostic, prognostic, predictive, or monitoring roles depending on their intended “context of use”[6]. In general, discovery-stage candidates should be evaluated along a translational continuum that includes analytical validation (reliable and reproducible measurement), clinical validation (replication of associations with relevant clinical outcomes), and finally clinical utility (demonstrated benefit for prognosis, stratification, or treatment decision-making). Within this framework, pan-cancer analyses can be particularly informative at the discovery stage by assessing the robustness and tumor-type specificity of biomarker signals and by prioritizing cancer types for focused validation and mechanistic studies.

6. Liu H, Karsidag I, Golin R, Wu G. Bridging Discovery and Treatment: Cancer Biomarker. Cancers. 2025;17(22):3720. http://doi.org/10.3390/cancers17223720 **Comment 2:** For the results sections that rely heavily on TCGA analyses, the overall workflow is acceptable and quite standard, but the manuscript treat TCGA almost as a neutral truth source, which is risky. Many readers already know this, but it still need to be said explicitly. It would help to situate the approach within prior TCGA-based efforts, such as TCGA-Based Analysis of Oncogenic Signaling Pathways Underlying Oral Squamous Cell Carcinoma or Immune prognostic model for glioblastoma based on the ssGSEA enrichment score, just to show that the strategy follow an established analytical tradition. At the same time, this is exactly where a more critical tone is needed. Bulk transcriptomic resources come with built-in biases, in sample composition, stromal contamination, batch effects, and clinical heterogeneity, and ignoring this weaken the interpretation of differential expression, immune infiltration, and pathway enrichment. A brief but explicit acknowledgement, supported by “Genetic Expression in Cancer Research: Challenges and Complexity” (2024) and “Technical and Biological Biases in Bulk Transcriptomic Data Mining for Cancer Research” (2025), would go a long way toward making the conclusions feel more measured and trustworthy.
**Response 2:** Thank you for your advice. We added the following descriptions in study limitations section and cited these studies: First, our TCGA-based analyses follow an established and widely adopted strategy in cancer bioinformatics, and Similar TCGA-driven frameworks have been applied to dissect oncogenic signaling programs in oral squamous cell carcinoma and to construct ssGSEA-based immune prognostic models in glioblastoma, supporting the methodological tradition underlying our approach[41, 42]. But bulk transcriptomic resources are not neutral truth sources and inherently contain both technical and biological biases[43, 44]. Therefore, our TCGA-based results should be interpreted as associative and hypothesis-generating rather than causal, and key observations require validation in independent cohorts and orthogonal experimental systems.

41.	Gao X, Liu D, Liu J, Wang R. TCGA-based analysis of oncogenic signaling pathways underlying oral squamous cell carcinoma. Oncology and Translational Medicine. 2024;10(2):87-92. http://doi.org/10.1097/ot9.0000000000000025

42.	Okamoto T, Mizuta R, Demachi-Okamura A, Muraoka D, Sasaki E, Masago K, et al. Immune prognostic model for glioblastoma based on the ssGSEA enrichment score. Cancer Genet-Ny. 2025;294-295:32-41. http://doi.org/10.1016/j.cancergen.2025.03.005

43.	Liu H, Li Y, Karsidag M, Tu T, Wang P. Technical and Biological Biases in Bulk Transcriptomic Data Mining for Cancer Research. J Cancer. 2025;16(1):34-43. http://doi.org/10.7150/jca.100922

44.	Liu H, Guo Z, Wang P. Genetic expression in cancer research: Challenges and complexity. Gene Reports. 2024;37:102042. http://doi.org/10.1016/j.genrep.2024.102042
**Comment 3:** When moving into the functional and immune-related analyses, the story become interesting but also a bit scattered. The immune microenvironment results are extensive, yet the biological implications are not always clear, and the paper sometimes describe correlation without really slowing down to explain what that might mean in a tumor context. I think the authors should lean more into interpretation here, especially where PRDX3 appear linked to immune modulation, checkpoint markers, or stromal features. Even a few sentences per subsection that ask what this could imply for therapy response or resistance would make the data feel less like a catalogue and more like a hypothesis-generating framework. here you should mention review paper "Identification of new immune target and signaling for cancer immunotherapy".
**Response 3:** Thank you for your advice. We agree that the functional and immune-related results, while comprehensive, could be read as a catalogue of correlations without sufficient biological interpretation in tumor context. In the revised manuscript, we reorganized the immune-related Results into a clearer narrative (TMB/MSI → ESTIMATE immune/stromal scores, immune cell infiltration, immune checkpoint/co-stimulatory genes, chemokines/receptors) and added brief interpretive sentences at the end of each subsection. Specifically, we now discuss what PRDX3-associated immune patterns could imply for immune activation vs. immune suppression/exclusion, potential adaptive immune resistance (checkpoint upregulation), and plausible links to immunotherapy response or resistance, while keeping the conclusions appropriately hypothesis-generating. We also cited the study.

24. Narote S, Desai SA, Patel VP, Deshmukh R, Raut N, Dapse S. Identification of new immune target and signaling for cancer immunotherapy. Cancer Genet-Ny. 2025;294-295:57-75. http://doi.org/10.1016/j.cancergen.2025.03.004

**Comment 4:** The experimental validation in KIRC is a strength, but the discussion stop short of translating this into a broader drug development perspective. Biomarkers do not live in isolation, and the manuscript would benefit from explicitly addressing how PRDX3-associated pathways, particularly the PPAR axis, could inform therapeutic targeting, patient selection, or combination strategies. In this context, citing and briefly discussing “Fast, tracking drug development with biomarkers and companion diagnostics.” would fit very naturally and help bridge the gap between molecular mechanism and clinical application. This does not require overclaiming, just a clearer statement of how such a biomarker could realistically be used.
**Response 4:** Thank you for your advice. We added the following descriptions in the discussion: From a translational and drug-development standpoint[40], this result may support PRDX3 as a plausible stratification biomarker in ccRCC, and our enrichment results implicate metabolic programs including the PPAR signaling axis. PPAR activity has been linked to metabolic reprogramming, antioxidant defenses, and immunosuppressive microenvironmental features that may contribute to therapeutic resistance; therefore, PRDX3–PPAR co-activation patterns raise a testable hypothesis that PRDX3 could help identify subsets of patients more reliant on lipid-metabolic/redox pathways and potentially inform pathway-guided targeting or rational combinations.

40. McBrearty N, Bahal D, Platero S. Fast-tracking drug development with biomarkers and companion diagnostics. Journal of Cancer Metastasis and Treatment. 2024;10:3. http://doi.org/10.20517/2394-4722.2023.134 **Comment 5:** Finally, the discussion and future outlook feel a bit conservative given how much data is presented. With deeper insight into tumor biology, cancer treatment is clearly moving toward personalized and precision approaches, especially those targeting the tumor immune microenvironment and specific molecular dependencies. I encourage the authors to end on a more forward-looking note, connecting their findings to emerging therapeutic paradigms and the idea that PRDX3-related vulnerabilities could eventually support tailored intervention strategies. Referencing “Overview of perspectives on cancer, newer therapies, and future directions” would help frame this vision and signal that the work is aligned with where the field is heading, even if much validation still remain to be done.

**Response 5:** Thank you for your advice. We revised the descriptions as follows: Cancer therapy is increasingly moving toward precision strategies that integrate tumor-intrinsic vulnerabilities with the immune microenvironment[45], and our findings nominate PRDX3 as a hypothesis-generating marker of redox–metabolic state (including PPAR-related programs) that could eventually support patient stratification and rational combination concepts. These translational implications remain preliminary and will require validation in treatment-annotated cohorts plus mechanistic and spatial/single-cell studies to confirm actionable PRDX3-linked dependencies.

45. Joshi RM, Telang B, Soni G, Khalife A. Overview of perspectives on cancer, newer therapies, and future directions. Oncology and Translational Medicine. 2024;10(3):105-9. http://doi.org/10.1097/ot9.0000000000000039

**Response to Reviewer #2:**

This study presents an interesting multi-omics investigation into the role of PRDX3 across 33 cancer types, with a specific focus on Kidney Renal Clear Cell Carcinoma (KIRC). While the bioinformatic integration is a clear strength, the manuscript requires additional experimental depth and technical clarification to support the authors’ mechanistic claims.
**Comment 1:** Please add recent statistics regarding kidney cancer prevalence and survival rates in the Introduction to provide a clearer background on the disease’s impact.

**Response 1:** Thank you for your advice. We have added the following descriptions: Kidney cancer remains a substantial global health burden. In 2022, GLOBOCAN estimated 434,840 new kidney cancer cases and 155,953 deaths worldwide, ranking kidney cancer 14th for incidence and 16th for mortality. GLOBOCAN 2022 also suggests a ~1.37 million 5-year prevalence globally (i.e., people living within five years of diagnosis). Despite advances in management, outcomes remain stage-dependent[4]

4. Filho AM, Laversanne M, Ferlay J, Colombet M, Pi Eros M, Znaor A, et al. The GLOBOCAN 2022 cancer estimates: Data sources, methods, and a snapshot of the cancer burden worldwide. Int J Cancer. 2025;156(7):1336-46. http://doi.org/10.1002/ijc.35278
**Comment 2:** Correlation between PRDX3 expression and PPAR pathway markers via Western Blot is insufficient to claim a regulatory relationship.

**Response 2:** Thank you for this important point. We agree that a correlation between PRDX3 expression and selected PPAR pathway markers measured by Western blot does not demonstrate a regulatory (causal) relationship. In the revised manuscript, we have therefore tempered our wording throughout to avoid overinterpretation. Specifically, we now describe the PRDX3–PPAR connection as an association (or “link”) supported by (i) pathway enrichment results and (ii) concordant protein-level changes, rather than stating that PRDX3 “regulates” or “drives” PPAR signaling.
**Comment 3:** Clarify if the reduced migration/invasion observed in KIRC cells is a direct effect on motility or a secondary effect of decreased cell proliferation.

**Response 3:** Thank you for your advice. Our results suggested that PRDX3 overexpression was associated with upregulation of pro-apoptotic genes (Bax, BMF, Bim) and downregulation of cell-cycle/invasion-related genes (CCND1, CCL20, MMP9; Figs. 10E–F). Thus, the observed reduction in migration/invasion may be driven by both direct effects on invasive programs and indirect effects due to altered proliferation/viability. We have added these descriptions in the discussion.

**Comment 4:** The study notes that PRDX3 exhibits "dual roles" in tumor biology across different cancers. Please provide a table or supplementary figure clarifying which of the 33 cancers show PRDX3 as an oncogene/ an antioncogene.

**Response 4:** Thank you for your advice. The judgement of dual roles of PRDX3 was based on the correlation of PRDX3 with clinical prognosis in cancers. These results and their descriptions were presented in Fig 2 (Page 12, line 245-261). **Comment 5:** The authors should provide data on the intracellular ROS levels in KIRC cell models after PRDX3 manipulation.

**Response 5:** Thank you for your advice. We agree that direct measurement of intracellular ROS after PRDX3 manipulation would strengthen the mechanistic interpretation. At present, we have not included ROS staining/quantification data in the manuscript, which we acknowledge as a limitation. We have therefore revised the Discussion to explicitly state that the redox link is inferred from PRDX3 biology and downstream phenotypes (apoptosis/proliferation/invasion markers), and that future work will include direct ROS measurements to establish whether PRDX3-dependent phenotypes are mediated by altered oxidative stress. We added the following descriptions: We did not directly quantify intracellular or mitochondrial ROS following PRDX3 manipulation; therefore, the redox-mediated interpretation remains inferential and requires confirmation with ROS assays and rescue experiments in future studies
**Comment 6:** Please justify the choice of KIRC cell lines used. Are these lines representative of the "high-methylation" or "low-methylation" groups identified in your TCGA analysis?

**Response 6:** Thank you for your advice. For our functional experiments, KIRC cell lines were chosen based on confirmed clear-cell renal carcinoma origin and broad use in KIRC mechanistic studies, complementary biological backgrounds and feasibility and reproducibility in our laboratory. Therefore, our cell-line work was designed primarily to test whether PRDX3 manipulation can influence KIRC phenotypes, rather than to model TCGA methylation strata directly.
**Comment 7:** While the in vitro data supports the role of PRDX3 in KIRC, the lack of animal models makes it difficult to assess the tumor microenvironment and immune response mentioned in the conclusion.

**Response 7:** Thank you for your advice. We revised the conclusion about tumor microenvironment and immune response. We also added the study limitations about the anima models.
**Comment 8:** The authors state that 50 pg of protein was loaded for Western blot analysis. Standard protocols typically employ 20 - 50 µg. Could the authors clarify if this is a typographical error? If 50 pg was indeed the amount used, please provide details on the high-sensitivity detection system or ultra-pure recombinant protein used to achieve a signal at this level.

**Response 8:** Thank you for your advice. We confirm that “50 pg” was a typographical error in the Methods section. The correct amount of total protein loaded per lane for Western blotting was 50 µg (not pg), which is consistent with standard protocols. We have corrected this unit in the revised manuscript**.
Comment 9:** The English needs improvement, and there are some errors. Be careful of abbreviations.

**Response 9:** Thank you for your advice. We have polished our manuscript and revised grammar errors. We also checked the abbreviations and listed all abbreviations in the S1 Tale.

**Response to Reviewer #3:**

**Comment 1:** Why do authors particularly choose KIRC not any other cancer type cell lines that are more significant in tumor microenvironment? Justify.
**Response 1:** Thank you for your advice. We selected KIRC for experimental validation based on a pre-specified integrative prioritization rather than convenience alone. Across our pan-cancer analyses, KIRC consistently showed: (i) significant PRDX3 dysregulation in tumor vs. normal tissues, (ii) robust clinicopathological associations, (iii) diagnostic potential in ROC-based evaluation, and (iv) consistent prognostic relevance across multiple clinically meaningful endpoints (OS, PFI, and DSS). Importantly, PRDX3 remained a significant factor in multivariable models in KIRC, supporting its potential independent prognostic value. We added the reason descriptions in the results.
**Comment 2:** How the findings can be clinically applied?
**Response 2: Thank you for your advice.** From a translational and drug-development standpoint, our result may support PRDX3 as a plausible stratification biomarker in ccRCC, and our enrichment results implicate metabolic programs including the PPAR signaling axis. PPAR activity has been linked to metabolic reprogramming, antioxidant defenses, and immunosuppressive microenvironmental features that may contribute to therapeutic resistance; therefore, PRDX3–PPAR co-activation patterns raise a testable hypothesis that PRDX3 could help identify subsets of patients more reliant on lipid-metabolic/redox pathways and potentially inform pathway-guided targeting or rational combinations. We added these descriptions in the discussion.
**Comment 3:** Fig.2 represent the same information so except Fig.2A others can be moved to supplement file.
**Response 3:** Thank you for your advice. Figure 2A presented the results of cox regression and K-M analysis in four clinical outcomes, which are qualitative. And Figure 2B-2E presented the results of cox results with HRs and 95%CI, which are quantitative. Moreover, the journal has no limitations for the number of figures. We just presented all results as many as we can.
**Comment 4:** Clarify the term TBM whether it is TMB or TBM
**Response 4:** Thank you for your advice. It should be TMB (Tumor Mutational Burden). We have revises this.
**Comment 5:** Fig. 4, Fig. 6, Fig.7 results does have much correlation with KIRC. Justify their importance or move them to supplement file.
**Response 5:** Thank you for your advice. Figures 4, 6, and 7 are retained to establish the pan-cancer tumor-microenvironment context of PRDX3 and to show that PRDX3–TME associations are tumor-type dependent rather than universal. This is important because it motivates why a single cancer type requires focused validation, and it demonstrates that KIRC is not chosen arbitrarily but sits within a broader landscape where PRDX3 shows meaningful TME/immune-gene associations. In KIRC specifically, PRDX3 shows non-trivial correlations with multiple immune-related modules (e.g., checkpoint-related genes such as CD274 in Fig. 6B; selected chemokines/receptors in Fig. 7A–B; and TME score associations in Fig. 4C–R), supporting biological plausibility for selecting KIRC for downstream validation.
**Comment 6:** Give a brief description of peroxisome proliferator-activated receptor signaling pathway in KIRC.
**Response 6:**Thank you for your advice. We have added a brief description in the revised Discussion to contextualize the enrichment result. Briefly, the PPAR pathway is mediated by ligand-activated nuclear receptors (PPARα, PPARβ/δ, and PPARγ) that heterodimerize with RXR and bind PPAR response elements to regulate transcriptional programs controlling fatty-acid uptake/transport, lipid storage, and mitochondrial/peroxisomal fatty-acid oxidation, with additional roles in inflammation-related signaling.
**Comment 7:** Give a pictorial representation of the identified pathway mechanism.

**Response 7:** Thank you for your advice. We have added a We have added a schematic diagram to provide an integrated, hypothesis-generating model summarizing our findings (S3_Fig.tif).
